# Comparative transcriptome meta-analysis of *Arabidopsis thaliana* under drought and cold stress

**DOI:** 10.1371/journal.pone.0203266

**Published:** 2018-09-07

**Authors:** Rinku Sharma, Garima Singh, Sudeepto Bhattacharya, Ashutosh Singh

**Affiliations:** 1 Department of life Sciences, Shiv Nadar University, Gautam Buddha Nagar, Uttar Pradesh, India; 2 Department of Mathematics, Shiv Nadar University, Gautam Buddha Nagar, Uttar Pradesh, India; National Taiwan University, TAIWAN

## Abstract

Multiple environmental stresses adversely affect plant growth and development. Plants under multiple stress condition trigger cascade of signals and show response unique to specific stress as well as shared responses, common to individual stresses. Here, we aim to identify common and unique genetic components during stress response mechanisms liable for cross-talk between stresses. Although drought and cold stress have been widely studied, insignificant information is available about how their combination affects plants. To that end, we performed meta-analysis and co-expression network comparison of drought and cold stress response in *Arabidopsis thaliana* by analyzing 390 microarray samples belonging to 29 microarray studies. We observed 6120 and 7079 DEGs (differentially expressed genes) under drought and cold stress respectively, using Rank Product methodology. Statistically, 28% (2890) DEGs were found to be common in both the stresses (i.e.; drought and cold stress) with most of them having similar expression pattern. Further, gene ontology-based enrichment analysis have identified shared biological processes and molecular mechanisms such as—‘photosynthesis’, ‘respiratory burst’, ‘response to hormone’, ‘signal transduction’, ‘metabolic process’, ‘response to water deprivation’, which were affected under cold and drought stress. Forty three transcription factor families were found to be expressed under both the stress conditions. Primarily, WRKY, NAC, MYB, AP2/ERF and bZIP transcription factor family genes were highly enriched in all genes sets and were found to regulate 56% of common genes expressed in drought and cold stress. Gene co-expression network analysis by WGCNA (weighted gene co-expression network analysis) revealed 21 and 16 highly inter-correlated gene modules with specific expression profiles under drought and cold stress respectively. Detection and analysis of gene modules shared between two stresses revealed the presence of four consensus gene modules.

## Introduction

Abiotic stress severely affects both physical and biochemical properties of plant cells, which then eventually alter survival and productivity. Abiotic stresses are one of the major causes of meagre plant growth and reduced crop yields globally. In most of the plant species, >50% growth reduction was observed due to abiotic stress [1]. In agricultural fields, plants have to encounter more than one stress simultaneously and try to acclimate to changing the climate. They have evolved several physiological, molecular and metabolic mechanisms that eventually leads to stress tolerance by achieving a homeostatic state [2–4]. Since, the stress adaptation mechanisms are largely unknown, elucidating these tolerance mechanisms is essential to accelerate plant adaptability to natural field conditions in order to enhance their growth and yield.

A huge amount of transcriptomic data is available for plants exposed to various abiotic stresses. Comparison of the transcriptomic data of plants exposed to individual and combined stresses may explain the molecular mechanisms behind the cross-talk between stresses. A meta-analysis is a promising approach which can be adapted to perform such comparison. It will help in identifying the biological processes activated in a specific stress. There are several studies on single stress conditions, which do not provide enough information about expression profiles of stress-responsive genes in multiple stress conditions. Recent investigations on multiple stress-induced biological networks received much attention[5–8]. Additionally, comparison of molecular profiles of an organism under different stresses would make it possible to identify the conserved stress mechanisms [5–8]. Gene co-expression networks study is becoming increasingly popular as one of the approaches to identify sets of interacting genes. The co-expression networks built from plant transcriptome data have been analyzed to unravel the stable co-expression relationships across distinct sets of experimental data [5,7,9].

*Arabidopsis thaliana* is a well-studied model plant organism. It has extensive biological knowledge base and resources including complete genome sequence and the highest number of microarray studies have been performed on *A*.*thaliana*. Therefore, meta-analysis has been performed by integrating individual *A*.*thaliana* stress microarray dataset, to understand the expression pattern of stress-responsive genes and molecular pathways in multiple stress conditions. Meta-analysis is helpful in understanding the common and dissimilar pathways as well as stress-responsive genes affected under multiple stress condition.

In the present study, a comprehensive meta-analysis was performed on *A*.*thaliana* microarray-based transcriptomic dataset for drought and cold stress. The analysis revealed unique as well as shared molecular components in drought and cold stress conditions. Additionally, among common genes, most of them showed conserved expression pattern and few showed reverse expression pattern which gives shreds of evidence about the molecular pathways functional during stress tolerance. Gene ontology (GO) enrichment analysis and GO profiles comparison was also performed to find shared and unique biological processes and molecular functions. Further, co-expression network analysis has also been studied, which clustered the differentially expressed genes to highly correlated gene modules with specific expression patterns, thus illustrating the framework of stress transcriptome. Altogether, the present analysis provides evidences about common and unique stress mechanism components under cold and drought stress in *A*.*thaliana*.

## Methods

### Data collection, curation and DEG finding

Microarray data were downloaded from NCBI Gene Expression Omnibus (platform accession number, GPL198) and EBI ArrayExpress Archive in March 2017.Each dataset contains more than 22,500 probesets representing approximately 24,000 genes (http://www.ncbi.nlm.nih.gov/geo/query/acc.cgi?acc=GPL198). For data collection, the NCBI GEO functional genomics repository was queried considering the Platform: GPL198. Experiments under this platform were searched using keywords, “cold stress” AND “Arabidopsis thaliana” [organism]; “drought stress” AND “Arabidopsis thaliana” [organism]. The ArrayExpress database of functional genomics experiments was mined (http://www.ebi.ac.uk/arrayexpress/) using keywords, “cold stress” / “drought stress” and filtered for “Arabidopsis thaliana” [by organism], “rna assay”, “array assay” [by experiment type] and “Affymetrix GeneChip *Arabidopsis* Genome [ATH1-121501] [by array]. The final dataset had 29 series, comprising of 241 and 149 Affymetrix *A*.*thaliana* arrays related to drought and cold stress respectively (S1 Table).

The dataset was normalized using GCRMA R package[10] and outlier samples were detected using the arrayQualityMetrics[11]R package. Arrays that failed any of the three outlier tests or not following two class formats (i.e. control and stress) were excluded from further analysis. Differentially expressed genes (DEGs) were determined by the function RPadvance in the Bioconductor package. RankProd[12] is a modified and extended function of Rank Product method proposed by Breitling and co-workers [13]. This method could assess the possible risk of biasness, as it is a non-parametric statistical test, derived from biological reasoning which detects items that are consistently ranked higher in a number of lists [12]. This method performed better than other methods, like- t-based hierarchical modeling and Fisher’s Inverse chi-square test, [14] and is utilized to directly combine multiple datasets into one meta-study[14]. The following parameters were used to generate the output of differentially expressed genes: a number of permutation tests = 250 and PFP (Percentage of false prediction) cut-off value = ≤0.01. The observed DEGS were matched to their loci based on annotation provided by array element mapping facility at TAIR portal for *A*. *thaliana* (http://www.Arabidopsis.org/portals/expression/microarray/microarrayElementsV2.jsp). Probes with no match or ambiguously matching multiple loci were discarded. Among multiple probes matching the same locus, the probe ID with highest fold change was retained.

### Gene Ontology (GO) enrichment analysis

Gene Ontology (GO) enrichment was performed using Singular Enrichment Analysis (SEA) tool of agriGO [15] using the default setting. Gene ontology profile analysis was used to compare DEGs of cold and drought stress with R package goProfiles version 1.34[16]. The significance of profile differences in annotation frequencies was tested for each gene ontology term, between level 4 for biological process, molecular function, and cellular component, using Fisher’s exact test followed by p-value adjustment for multi-testing, based on Holm-Bonferroni method. Transcription factors (TFs) annotation for *A*.*thaliana* were obtained from the database PlnTFDB[17] and analyzed for transcription factors potential targeted genes among common differentially expressed genes of cold and drought stress using AthaMap gene analysis tool[18,19].

### Gene co-expression network analysis and consensus module detection

Pair-wise gene expression Pearson correlation across all the samples was calculated to generate a similarity matrix, which served as an input for generating the stress-specific co-expression networks (using R/WGCNA version 1.34) [20]. Soft threshold, (β) 10 and 13 for drought and cold respectively, has been identified to calculate adjacency, based on the criterion of approximate scale-free topology. To minimize effects of noise and spurious associations, the adjacencies transformed into Topological Overlap Matrix and were then converted into a dissimilarity matrix. Further, a hierarchical cluster tree was created, based on the dissimilarity matrix and gene co-expression modules were identified from the hierarchical cluster tree using dynamic tree cut method. This method identifies modules whose expression profiles are very similar. For this analysis, module size was 30, deepSplit was set at level 1 and tree mergecutHeight was 0.20. Such branches corresponded to modules having eigengenes with a correlation of 0.80 or higher. The differentially expressed genes common to drought and cold stress dataset were further investigated to find gene modules shared by gene co-expression network of drought and cold stress also known as consensus modules, with a setting as a soft threshold (β) 10 and module size 30. Further, consensus modules were compared with drought and cold global co-expression network gene modules for which calculated the overlaps of each pair of drought/cold-consensus modules and used the Fisher’s exact test to assign a p-value to each of the pair-wise overlaps. Then, differential consensus module eigengene network analysis was performed by comparing the connectivity and module structure of two networks based on the expression data of differentially expressed genes common to cold and drought stress dataset.

Further, module preservation statistical tests [21] were performed for determining which properties of a module in one reference network were preserved in a second (Test) network using the WGCNA modulePreservation function. The composite module preservation statistics Z_summary_ and medianRank was used to define preservation relative to a module of randomly assigned genes. The Z_summary_ summarized density and connectivity based preservation statistics where values 2>Z represented no preservation, 2< Z<10 represented weak to moderate preservation, and Z>10 represented strong preservation (Eq 1).

$$Z_{\mathrm{summary}}=\frac{Z_{\mathrm{density}}+Z_{\mathrm{connectivity}}}{2}$$ 1

The medianRank is a rank based statistics that rely on observed preservation statistics. It summarized medianRank: density and medianRank: Connectivity. A module with lower median rank exhibit higher observed preservation statistics than a module with a higher median rank (Eq 2).

$$\mathrm{medianRank}=\frac{\mathrm{medianRank}.\mathrm{density}+\mathrm{medianRank}.\mathrm{connectivity}}{2}$$ 2

The consensus modules detection method identified groups of genes with highly correlated expression profiles which could be represented by a single gene: the module eigengene (defined as the first right-singular vector of the standardized expression profile for each module) [22]. Eigengene network for cold and drought stress related common gene dataset was constructed where connection strength (adjacency) between eigengene (E) *I* and *J* was defined as (Eq 3):

$$a_{Eigen,IJ=\frac{1+cor(E_{I,}E_{J})}{2}}$$ 3

The correlation preservation between all pairs of consensus modules of cold and drought stress networks was considered, A_Eigen_^(Cold)^ and A_Eigen_^(Drought)^ are the adjacency matrices for dataset cold and drought as defined in Eq 3. Network preservation was commuted in which adjacencies are defined as (Eq 4):

$${Preserv}_{IJ}^{Drought,Cold}=1-\frac{\left|cor(E_{I}^{Drought},E_{J}^{Drought})-cor(E_{I}^{Cold},E_{J}^{Cold})\right|}{2}$$ 4

Where E_I_^(X)^ is the eigengene of the I-th module in the dataset X. Large values of Preserv_IJ_^Drought,Cold^ showed robust preservation among the two networks, of the correlation between module eigengenes I and J. The scaled connectivity which describes the average connection strength of I-th module with all other eigengenes of the preservation network is defined as (Eq 5):

$$C_{I}{Preservation}_{IJ}^{Drought,Cold}=1-\frac{\left\{\sum_{J\neq I}|cor(E_{I}^{Drought},E_{J}^{Drought})-cor(E_{I}^{Cold},E_{J}^{Cold})\right\}}{2(N-1)}$$ 5

Where N is the number of Module eigengenes. Density of the eigengene network D Preserv^(Drought,Cold)^ defined as the average scaled connectivity, is defined as (Eq 6):

$$(D{Preserv}^{\left(Drought,Cold\right)})=1-\frac{\left\{\sum_{I}\sum_{J\neq I}|cor(E_{I}^{Drought},E_{J}^{Drought})-cor(E_{I}^{Cold},E_{J}^{Cold})|\right\}}{2N(N-1)}$$ 6

Values of D close to 1, represent strong preservation of correlation between all the eigengene pairs across networks.

## Results and discussion

### Data collection and curation

**Fig 1:**
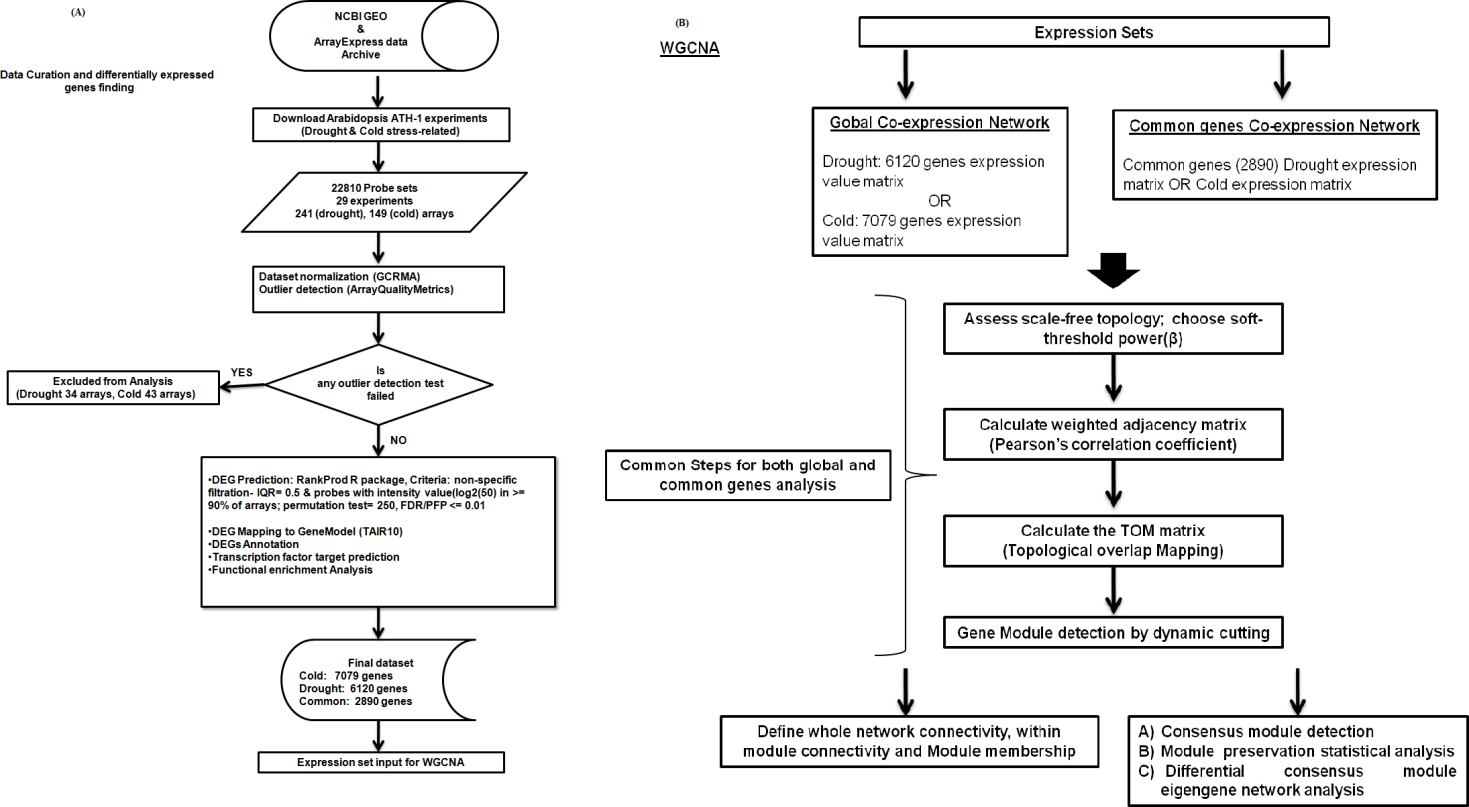
Workflow for data collection, curation and co-expression network analysis. **(A)** In total 29 series (26 series from NCBI-GEO and 3 series from ArrayExpress) comprising of 241 and 149 Affymetrix *A*. *thaliana* arrays related to drought and cold stress were used in the analysis. (B) Workflow describes the steps for co-expression network generation and consensus module detection.

A schematic workflow of the analysis from data collection and curation, differentially expressed genes prediction, network construction, to consensus module detection and characterization is described in Fig 1A and Fig 1B.

Publically available microarray experiments involving drought and cold stress from ATH1-121501 Affymetrix *Arabidopsis thaliana* Genome Array were collected (S1 Table). The raw data related to drought and cold stress were normalized by GCRMA approach and the relative quality of different samples within dataset was examined by ArrayQualityMatrics, R package. This R package performed- A) comparison among samples by checking the distance between samples, B) sample intensity distribution by boxplots and C) individual sample quality by MA plots. Sample failing any of the aforementioned statistics was discarded. Then, filtered datasets were used to detect differentially expressed genes under cold and drought stress by the RankProd method.

### Comparison of differentially expressed genes under drought and cold stress

**Fig 2:**
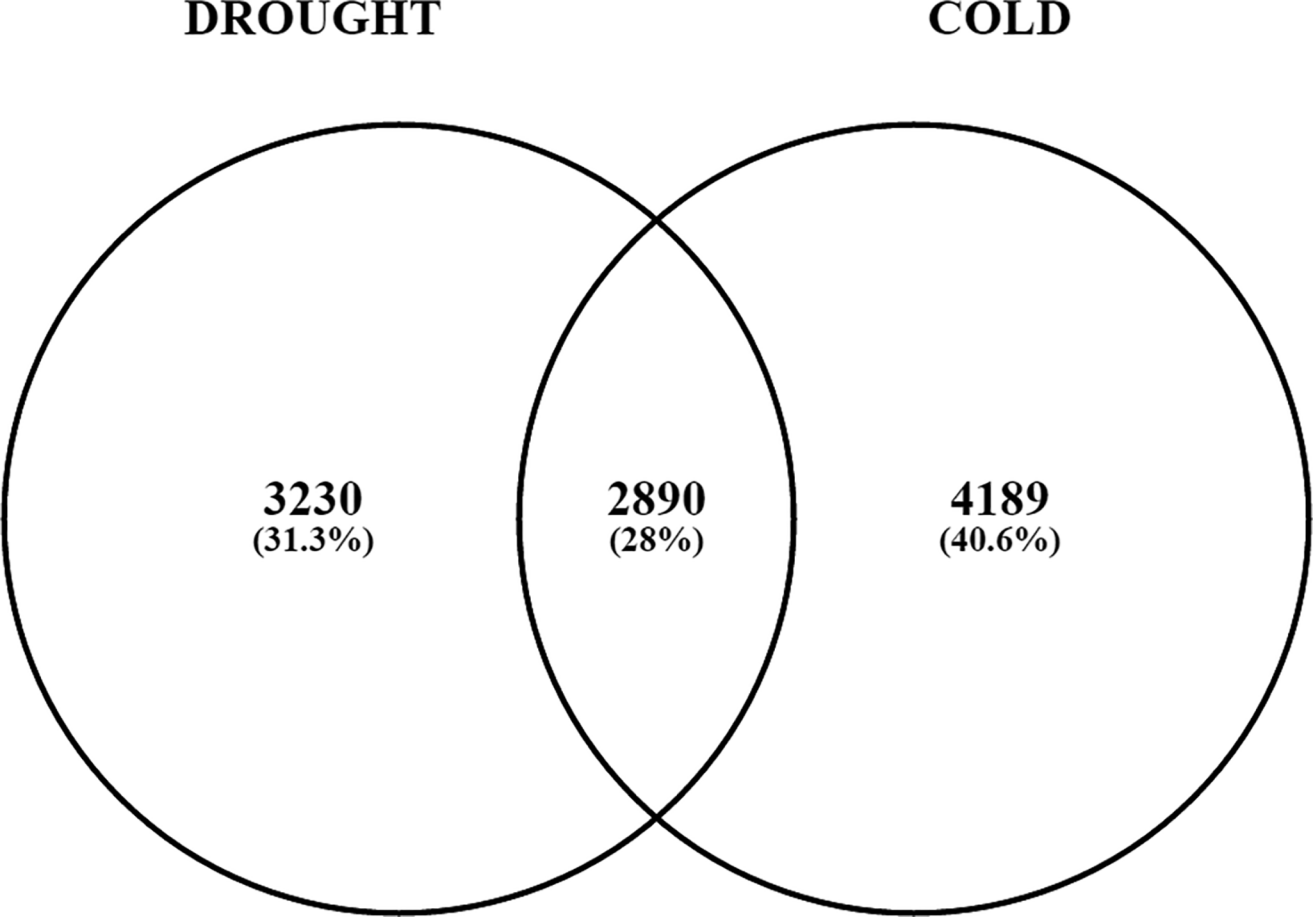
Number of unique and common differentially expressed genes (DEGs) found in *A*.*thaliana* under cold and drought stress. Total number of genes is shown in bold, below which are the percentages of genes.

From 29 series, 6350 and 7210 differentially expressed probes, mapped to 6120 and 7079 gene models in *A*.*thaliana* were identified with PFP (percentage of false prediction) ≤0.01, under drought and cold stress respectively. Here, we report, 41% and 31% genes unique to drought and cold stress respectively (Fig 2). In case of drought condition, 52% of DEGs were up-regulated and 48% were down-regulated whereas in case of cold stress, 48% were up-regulated and 52% were down-regulated (S2 Table). DEGs common to both the stresses were found to be 2890. Most of the genes showed conserved expression pattern (72% or 2083) with 1084 up-regulated and 999 down-regulated in both drought and cold stresses (S3 Table).Hence, molecular profiles of common DEGs suggests that common molecular pathways are altered in a similar manner in response to both the stresses. In a group of genes with non-conserved expression pattern, the proportion of genes showing down-regulation in drought and up-regulation in cold stress is 356 (or 44% of 807) and up-regulation in drought and down-regulation in cold stress is 451 (or 56% of 807). Further, estimated percentage of false prediction (PFP) values of common DEGs were compared, and it was observed that 63% (1828) and 64% (1860) genes under drought and cold stress respectively were highly significant (PFP <0.0001) which included ~36% (1029/2890) of stress-related genes up-regulated under both the stresses (S3 Table).

Differentially expressed genes (DEG) were compared with literature-derived cold and drought dataset genes. Approximately 70% and 37% DEGs of meta-analysis (under drought and cold stress respectively) were also reported previously in experimental studies (S4 Table). Among common DEGs (cold and drought stress), 843(29%) genes were also found in both cold and drought stress related published literature. There were seven genes (up-regulated) which were also reported [23] to be commonly up-regulated under both cold and drought stress (S5A and S5B Table). Although the results were comparable to individual published studies, our meta-analysis also identified many new stress-responsive genes (Table 1).

**Table 1:** Comparison of meta-analysis result with individual expression studies literature.

Stress	Common to literature and Analysis	Unique to Analysis
**Cold**	2604	4475
**Drought**	4319	1801
**Common(cold & drought)**	843	2047

Rest *et al*.[24]had used a similar approach of meta-analysis to integrate microarray studies on water stress in *A*.*thaliana* and compared results with the published literature. The study reported that meta-analysis was able to identify genes with consistent overall expression patterns, and also rejected genes with inconsistent expression across individual datasets. It shows the reliability and strength of present meta-analysis to identify additional responses that were not identified by conventional approaches.

The mean expression change in response to both the stresses was >2(2.17 and 2.24 fold change under cold and drought stresses respectively). The percentage of DEGs with fold change >2 was higher in cold stress (~55%), most of which were down-regulated and lower in drought stress (52%), but under drought stress majority was up-regulated. There were 16 and 6 genes with fold change >10 under drought and cold stresses respectively (S2 Table). Notably, three genes showed >20 fold change expression in cold stress with AT2G34620 gene model annotated as *MTERF10* (mitochondrial transcription termination factor family protein) was under expressed by 23.78 folds. MTERFs are required for accurate organelle gene expression [25]. Zhao *et al*. in 2014[26] reported that down-regulation of *MtERF* enhanced tolerance of *Medicago truncatula* to freezing by up-regulating down-stream genes. This gene was found to be overexpressed in drought (5.7 fold changes). AT4G28270 gene model annotated as RING membrane-anchor 2 (*ATRMA2*, *RMA2*) was the second top DEG which was up-regulated in both stress conditions (22.5 and 2.4 fold changes in cold and drought stress respectively). *ATRMA2*, *RMA2* are E3 ubiquitin ligase that plays a key role in regulating cellular expression level of *ABP1* (Auxin binding protein 1)[27]. Another gene that was highly up-regulated in both the stresses is *EXPA8*(21.5 and 16.8 fold changes in cold and drought stress respectively) that promotes cell wall loosening by inducing pH-dependent cell wall extension and stress relaxation[28].

### Shared and unique responses under cold and drought stress

The present study suggested that *A*.*thaliana* shows shared and unique molecular responses for survival under multiple stress condition. It is important to identify these common and unique responses under cold and drought stress for understanding the cross-talk mechanism. Gene ontology profiles of DEGs in cold and drought stresses were statistically compared to assess the biological similarity and differences between the two DEGs list. We were able to identify 142 common significant gene ontology terms such as, ‘photosynthesis’, ‘respiratory burst’, ‘response to hormone’, ‘signal transduction’, ‘metabolic process’, ‘response to water deprivation’ etc (S6 Table).

Both stress factors affect the homeostasis of chemical signals at the apoplastic space such as Ca^2+^ and ROS[29]. Many signaling nodes like RBOH(AT1G64060), RLKs (AT3G17840, AT3G45860, and AT4G23210) and cell wall kinase (AT1G21250) were found to be expressed under both the stresses and play role in early signal perception and transduction[29]. Several mitogen-activated protein kinases (MAPK) which links the external stimuli to intercellular responses (MAPK) (AT1G05100, AT3G07980 and AT1G53570) were also shared by both stresses [30]. Genes such as calmodulins (AT1G76650,AT3G51920 andAT2G26190), calcium-dependent protein kinases(CDPKs) (AT3G50530, AT1G18890), glutathione S-transferase (AT3G43800, AT1G10360) and ascorbate peroxidase (AT4G09010) which helps in maintaining ROS homeostasis were also found to be over-expressed under both the stress condition[31,32].Stress-specific genes were also identified, for example, COR (Cold-regulated) genes: *COR47* (AT1G20440), *COR15B* (AT2G42530) and *COR15A* (AT2G42540) encoding cryo-protective polypeptides which enhances the cryo-stability of the plasma membrane [33].

### Comparison of transcription factor families’ abundance under drought and cold stress

**Fig 3:**
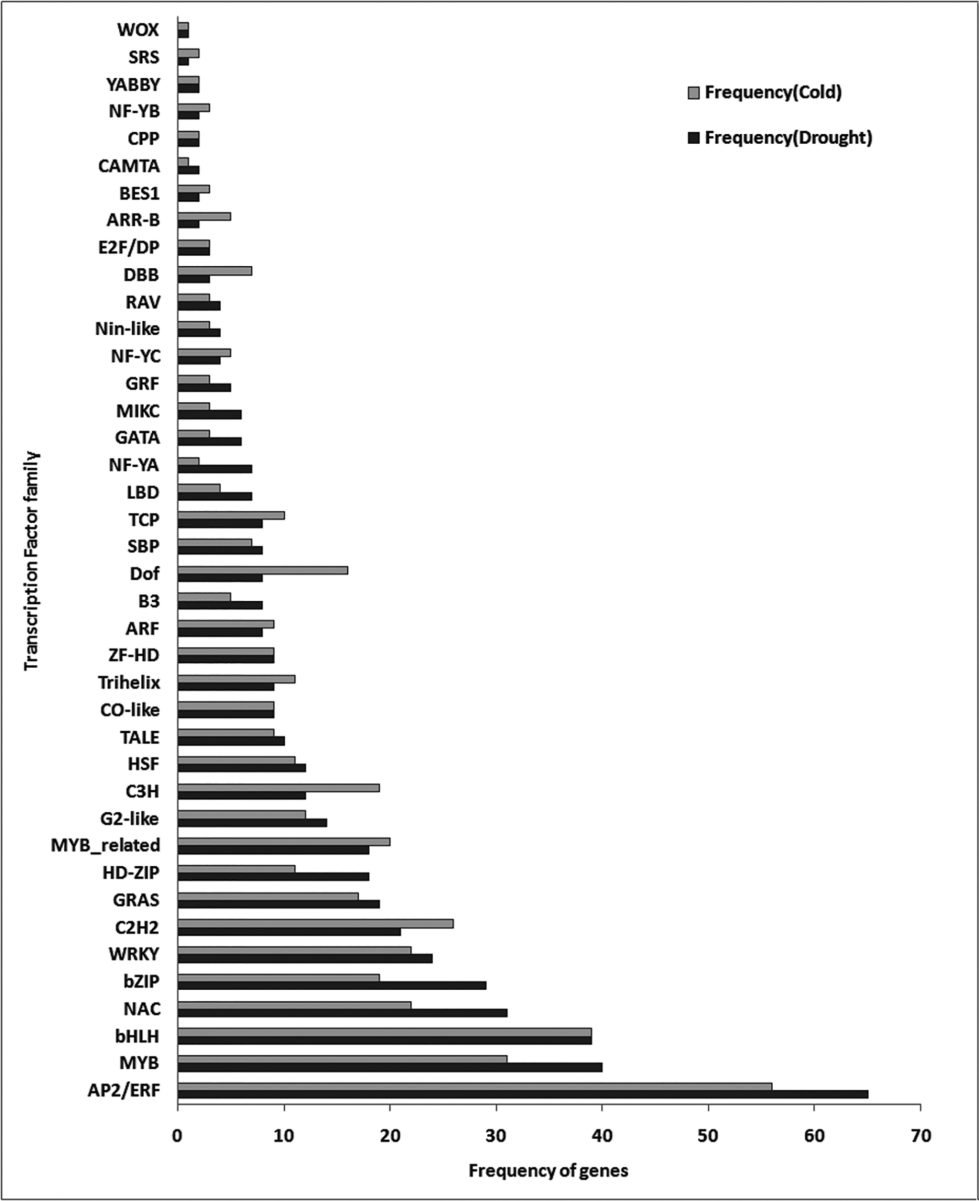
Abundance of transcription factors in transcription factor families expressed commonly under cold and drought stress. Grey bar: Number of transcription factors in each transcription factor family expressed under cold stress. Black bar: Number of transcription factors in each transcription factor family expressed under drought stress.

The identification of downstream regulators involved in multiple stress cross-talks such as transcription factors is important for targeted manipulation and adaptation of plants to stress combination. The favourable calibration of their expression has emerged as an effective strategy towards translation of scientific knowledge in crop plant improvement [34]. In the present study, we have identified 49 and 46 unique transcription factor families expressed specifically under cold and drought stress respectively, among which 43 families were commonly expressed (S7 Table) and most of them had the same kind of regulation under both the stress conditions (S3 Table). The abundance of common transcription factor families is higher in drought stress as compared to cold stress (S7 Table). WRKY, NAC, MYB, AP2/ERF, and bZIP were the most abundant transcription factor families under both the stresses (Fig 3). These transcription factor families are known for role in ABA-induced signaling pathways under cold and drought stress [35]. Both these stresses cause desiccation of the cell and osmotic imbalance and to combat this condition, in plants ABA biosynthesis is stimulated. Accumulation of ABA triggers several signaling pathways which ultimately helps the plant to regain homeostatic state; for an instance, under drought stress, in an ABA-dependent manner; bZIP, MYB and AP2/ERF transcription factor family interacts with ABRE, MYCRE/MYBRE or CRT/DRE elements in the promoter of stress genes and under cold stress, C2H2 transcription factor family members (SCOF and SGBF) follows ABA-dependent pathway to trigger expression of cold-regulated (COR) gene involved in imparting cold tolerance[36,37]. This is also proved by the transcription factor binding site analysis of common DEGs which showed that among common DEGs, 56% genes are regulated by AP2/ERF (326), bZIP(113), C2H2(372), MYB(379), NAC(1139) and WRKY(1) and most of these TFs target genes coding for proteins involved in ROS scavenging pathway, signaling, development and late responses to stress (S3 Table). For example, *ATCLH1* (encode Chlorophyllase 1) gene which is involved in plant damage control and modulation of balance between different plant defense pathways[38] is regulated by bZIP and NAC. Another set of genes, *APX4* (Ascorbate peroxidase4),AT5G51010 (Rubredoxin-like) and AT1G77370 (glutaredoxin) which are players of an enzymatic and non-enzymatic mechanism for scavenging free oxygen radicals[32,39,40]were found to be regulated by C2H2, MYB, and NAC transcription factor families. The signaling molecules like- protein tyrosine kinases, *CNX1* (calnexin 1), *ATRABG3B* (GTP-binding protein Rab7) and few more were targeted by AP2/ERF, C2H2, NAC, and bZIP transcription factor families. The members of MYB, NAC, and AP2/ERF transcription factor families were also found to target genes of development process such as *LEA* (late embryogenesis abundant protein) and *PLP9* (PATATIN-LIKE PROTEIN 9). The LEA protein also provides tolerance to dehydration which may be induced by freezing, saline conditions, or drying [41]. In literature, it has been reported that the transcription factor NAC gets induced by multiple abiotic stresses; it regulates plant growth and development including tolerance to several abiotic stresses [42]. Our results are comparable to published reports that NAC transcription factor regulates LEA and protein kinases which are marker genes in abiotic stress and ABA response pathway[43].

Genome-wide transcriptomic and microarray analyses have shown that many MYB proteins and MYB-binding element-containing genes are responsive to drought in *A*.*thaliana* and other plants [44]. MYB proteins regulate stomatal movement and are known to get down-regulated by drought stress. Its over-expression results in hypersensitivity to water deficiency [45]. It also acts as positive regulators of drought tolerance by activating the transcription of dehydration responsive genes, such as ERD(Early response to dehydration) [46,47]. MYB also acts as a negative regulator of freezing tolerance by suppressing the expression of *CBF1*[48]. A comparative list of the number of transcription factor members belonging to each transcription factor family is provided in the S7 Table. There were six transcription factor families found to be exclusively expressed during cold stress, EIL, GeBP, HB-other, M-type, NF-X1, and S1Fa-like. EIL is a primary transcription factor in ethylene signal transduction which regulates downstream genes to complete the ethylene response. It also regulates an innate immune receptor (FLS2) [49]. GeBP is a new class of unconventional Leu-zipper TF proteins which act as an antagonist in cytokinin pathway to negative feedback regulation on ARR genes and trigger the cytokinin response [50]. The NF-X1 transcription factor is a part of regulatory mechanisms, which safeguard major processes such as photosynthesis [51]. Whirly transcription factor family was expressed only under drought stress. It acts as an upstream regulator of drought stress-induced senescence [52].

### Gene co-expression network analysis

The primary aim of co-expression network analysis is to determine cluster or modules of densely inter-connected genes which can be analyzed by searching patterns in connection strength[53]. In the present study, the co-expression network analysis was performed by using transcriptome metadata collected from NCBI GEO, EBI ArrayExpress Archive (S1 Table). The similarity matrices generated from 207 and 106 filtered samples of drought and cold stresses respectively, were further processed to generate weighted co-expression network with scale-free topology by raising them to power β. Network construction for drought and cold stress resulted in 6,120 and 5,116 nodes connected by 12, 02,905 and 1, 45,476 edges respectively, under the threshold of 0.02. The global networks were further clustered into 21 and 16 modules for drought and cold stress respectively, using WGCNA package. The module of each DEG sets was indicated by following measures: 1. their whole network connectivity, kTotal, 2. the within module connectivity, kWithin, a measure of how well connected or co-expressed a given gene is, with respect to other genes in global network and in its module and 3. MM (Module Membership) for each gene in each module, a measure of module membership correlating its gene expression profile with the Module Eigengene (ME, the first principal component of a given module and can be considered a representative of the gene expression profiles in a module) (S8 Table).

**Fig 4:**
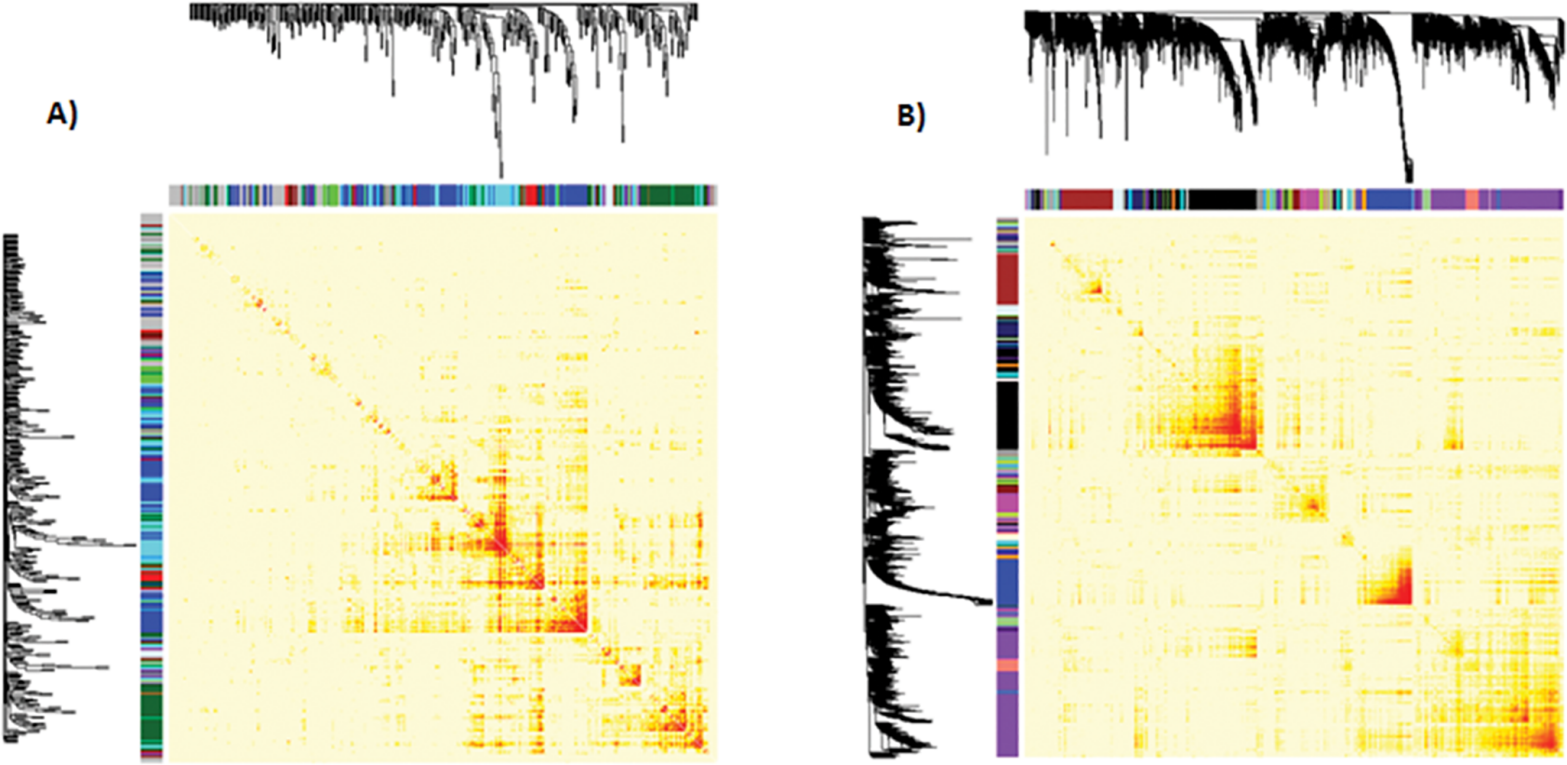
Dendrogram and heatmap of DEGs found under cold and drought stress in *A*. *thaliana*. The heatmap describes the Topological Overlap Matrix (TOM) for all DEGs used for co-expression network analysis. Color ranges from darker red to light red according to overlap i.e. higher to low and darker red color blocks along the diagonal are the modules. The gene dendrogram and modules (as different color bars) are also shown along the left side and the top.

The deep red color in the heat maps of co-expression networks illustrates high co-expression of DEGs within modules and less co-expression outside the module (Fig 4). The unsigned Pearson correlation was used; therefore all genes with same absolute correlation value were grouped into the same module. However, some gene modules showed significant enrichment of genes with the same kind of regulation (S9 Table). For example, the largest module, dark-green in color of cold stress co-expression network of size 1373 DEGs was enriched with 902 (~66%) down-regulated DEGs while the second largest module, blue in color of size 1291 DEGs was enriched with 777(~60%) up-regulated DEGs. Drought responsive 21 modules in the present study were compared against 5 modules of *A*.*thaliana* predicted by another group of researchers [7]. All the five modules were notably overlapped with modules in the present study. For example, the brown colored module found by Shaik and Ramakrishna[7] consists of 64 genes, out of which 45 (70%) were the part of magenta colored module of drought co-expression network and of blue module, 40% genes were overlapping with drought black module of present study (S10 Table).

To determine the stress-responsive modules of functionally similar genes, gene ontology (GO) enrichment analysis for all the predicted modules was performed. A number of significant terms with FDR< 0.05 were identified (S9 Table). The analysis showed a large difference in the number of significant GO terms compared to module size, in both cold and drought co-expression networks (S9 Table). For example, the cold module light green (module size 115) had 10 significant terms whereas grey60 module (module size 104) had 3 terms with FDR < 0.05. The drought module midnight-blue (module size 141) had 18 significant terms whereas salmon color module (module size 191) had 3 significant terms. Further analysis of these modules showed that cold module (grey60) and drought module (salmon) had a higher number of genes with unknown function and it was also observed that these genes had module membership >0.6 which indicates that these genes may have functions similar to other annotated genes.

Under cold stress, in the largest dark-green module, ~65% genes were under-expressed and found to be predominantly enriched with genes belonging to photosynthesis and carbon metabolism pathways. This module also had genes related to MAPK cascade, which implies that the module may serve as a negative regulator as well as a positive regulator up to some extent. In the second largest blue module, ~60% genes were over-expressed and the top functional term was ‘response to abiotic stimulus’ (GO:0009628) followed by ‘signal transduction’ (GO:0007165) (S11 Table) which suggests the role of this module in sensing the stress and relaying the signal to down-stream genes for action. The blue module had a larger number of TFs than the dark-green (89 compared to 69 TFs) (S8 and S9 Table). But it was made up of less number of genes than the dark-green module. Majority of blue module TFs were from AP2/ERF, HSF, and MYB transcription factor families while the dark-green had a higher number of MYB, bHLH, and NAC transcription factor families (S8 Table).

Under drought stress first two largest modules (namely, purple and black) had less than 40% down-regulated genes and were enriched with genes related to ‘response to stress’ and ‘metabolic process’ but the number of genes was higher in the black module. These modules also had Amylase, Invertase, SPS, and sugar transporter genes which indicated the availability of low-molecular-weight carbohydrates during drought stress [54].It was found that the transcription factor abundance was higher in purple module (99 TFs as compared to black module 78 TFs) and both the modules contain 25 common unique transcription factor family members including-NAC, MYB, AP2/ ERF, bZIP, and bHLH transcription factor families (S8 and S9 Tables) which are involved in ABA signaling pathway and stomata closure [35,37]. It indicates the role of purple and black modules in drought stress management by regulating stomata closing and ABA signaling pathways for reducing water loss, thereby minimizing photosynthesis activity and shifting to other metabolic pathways to meet energy demands.

### Module preservation statistical analysis

**Fig 5:**
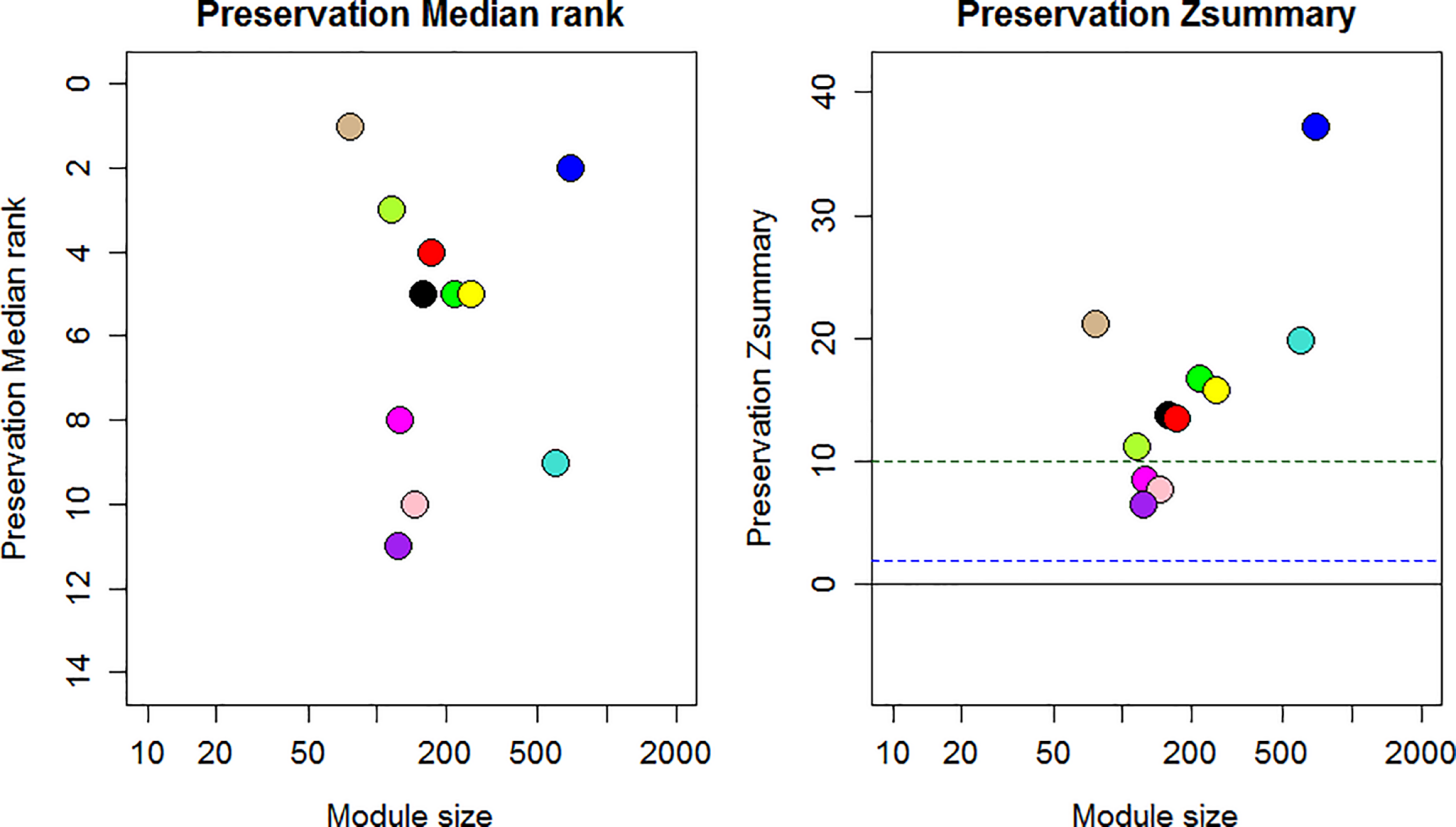
The medianRank and Zsummary statistics of module preservation of cold modules in drought modules (y-axis) vs. module size (x-axis). In plot colored circles represents gene modules of cold common gene co-expression network. The black borderline represents no preservation (Z-score = 0), blue borderline represents very weak preservation limits (Z-score = 2). The region between blue borderline and green borderline represents weak to moderate preservation zone (10 < Z-score > 2) and above green borderline represent strong preservation (Z-score> 10).

In order to determine how well the gene modules in the cold stress dataset (reference network) were preserved and reproducible in the drought stress dataset (test network), module preservation statistical analysis was performed using a series of permutation tests for various density and connectivity based measures. Eight cold modules were shown to have well-defined drought counterparts (summary Z-score >10) and two cold modules: Pink and Purple have shown moderate preservation (10 < Z-score > 5 and higher preservation median rank) (Fig 5). A “toy” module of 1000 randomly assigned genes collected from all possible gene (“Gold”) also revealed evidence of preservation across species. The preserved modules genes were found to associate with biological processes: photosynthesis, stress acclimation process, response to abiotic stress and metabolic process, which generally altered during stress (S11 Table).

### Identification of consensus modules

**Fig 6:**
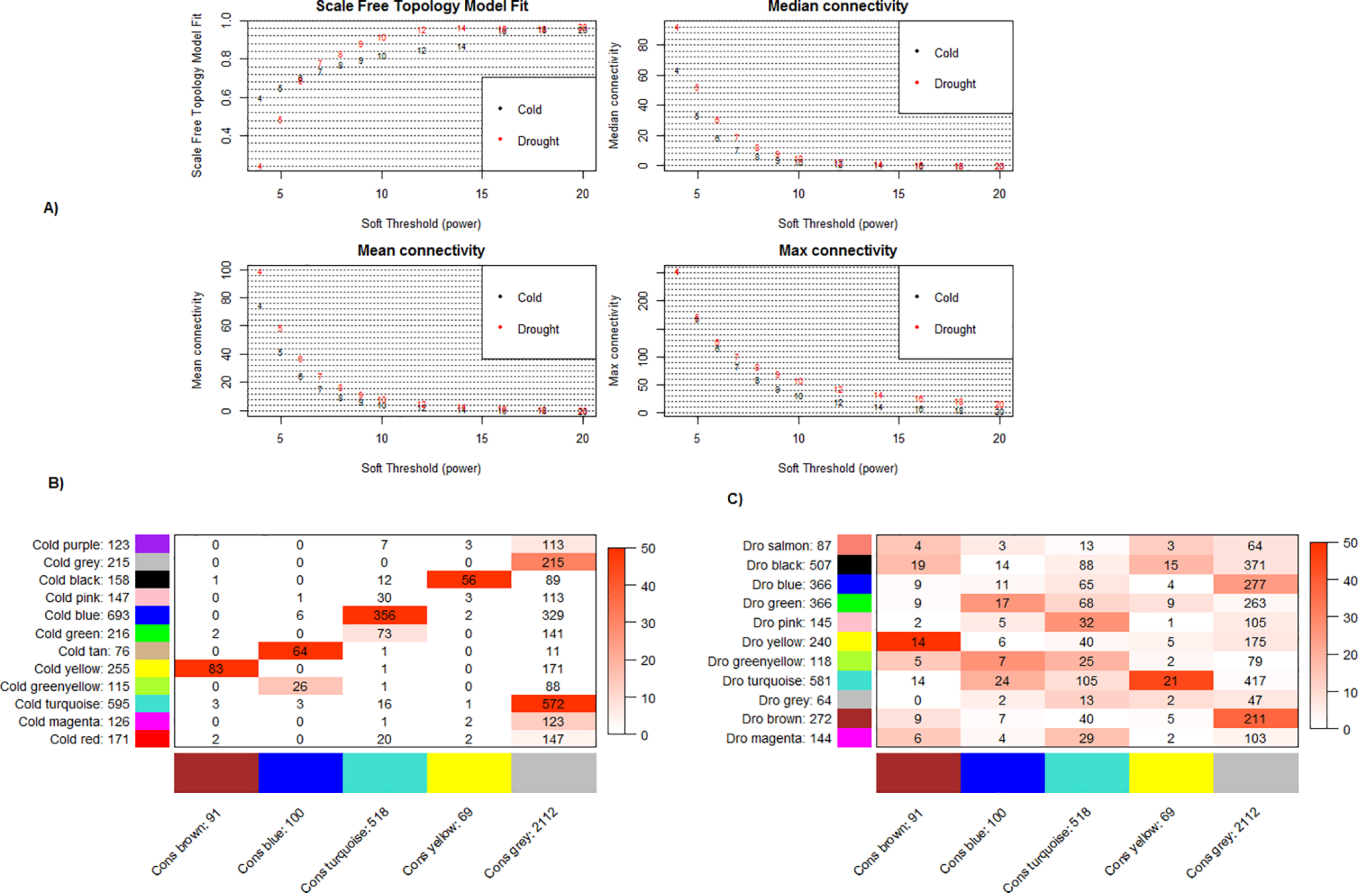
Consensus module detection and comparison. (A) The plots depicting various network indices (y-axes) as functions of the soft- thresholding power (x-axes). Numbers in the plots represents soft-thresholding powers. The plots demonstrate that 10 is the smallest soft-thresholding power at which approximate scale-free topology is accomplished for both sets as the various connectivity measures decrease sharply with increasing soft-thresholding power. (B) and (C) Relation between consensus modules and modules found individually in cold and drought expression set. The table represents the analogy between consensus modules and modules of cold and drought stress related global co-expression network based on the expression values of the common genes. Each row of the table represent individual stress specific module and each column represent one consensus module. Numbers in the table represent genes common between individual stress module and consensus module. The table coloring pattern represents the negative log of Fisher’s exact test p-value for the overlap of the two compared modules. The darker red color represents more significant overlap.

A consensus dissimilarity measure based on weighted average of the two correlation matrices was utilized in the hierarchical clustering algorithm for consensus module identification, with an objective to explore the group of genes with similar co-expression patterns and common, robustly defined modules in both the stresses. The consensus network of scale-free topology was obtained by using soft-threshold of 10, comprising of 4 consensus modules (Fig 6A). The genes which are not assigned to any of the modules were labeled as a grey color. The functional enrichment of the consensus modules included: “translation” (C1: Brown), “response to water deprivation and cold stress” (C2: Blue), “photosynthesis” (C3: Turquoise) and “defense response” (C4: Yellow). The full functional enrichment analysis and gene lists were given in S12 Table. A pair-wise Fisher exact test was performed to determine whether there is significant overlap between the consensus and the cold and drought specific gene modules. Fig 6B and 6C, represents the summary of the result in the form of color-coded tables illustrating good agreement between consensus modules and cold or drought specific modules, which reflects the facts that most cold modules are preserved in drought. All consensus modules showed significant overlap with their corresponding gene modules of stress-specific co-expression networks demonstrating akin nature of clustering pattern in cold and drought stress.

### Differential consensus module eigengene network analysis revealed highly preserved network structure

**Fig 7:**
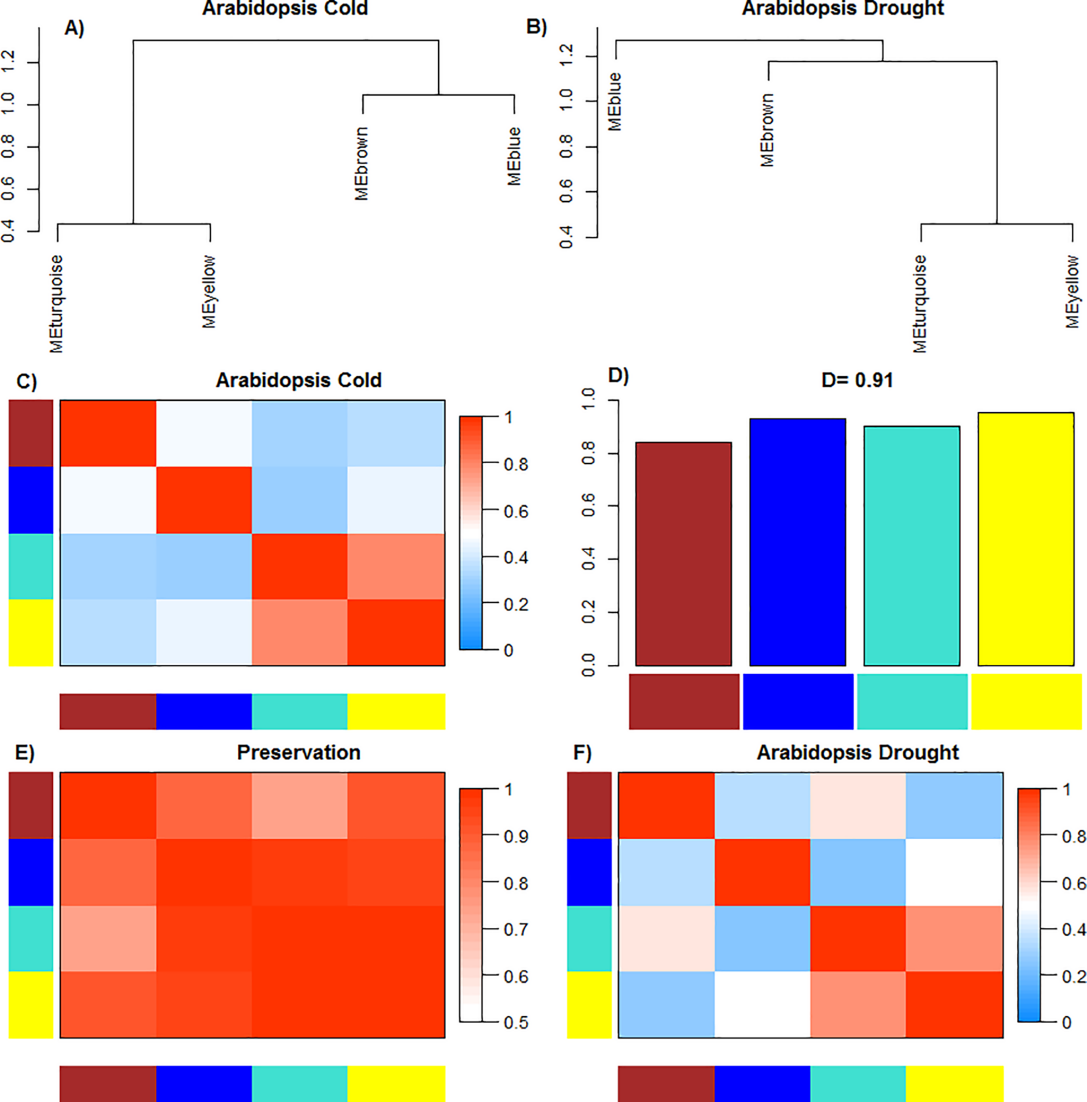
Differential consensus module eigengene analysis between cold and drought consensus module eigengene networks. Differential eigengene network analysis was used to address the strength of the correlation preservation for all eigengene pairs across the two networks: (A) and (B) Clustering dendrograms of consensus module eigengenes (ME) showing the presence of meta-modules as depicted by the presence of same major branching pattern in both cold and drought eigengene network dendrograms. (C) and (F) Heatmaps of eigengene adjacencies in each of the consensus eigengene networks for cold and drought dataset respectively. Each of the rows and columns represents an eigengene tagged by the consensus module color and within the heatmap, red color represents high adjacency and positive correlation, whereas blue represents low adjacency and negative correlation, as represented by the color legend. (D) Bar plot depicts the preservation measure for each consensus eigengene as the height of the bar (y-axis) where each colored bar corresponds to the eigengene of the associated consensus module. The high-density value D(Preserv^Drought,Cold^) = 0.91 indicates the high overall preservation between the two networks. (E) Heatmap representation of Adjacencies of the pair-wise preservation network (Preserv^Drought,Cold^); high values of (Preserv^Drought,Cold^) implies that there is a strong correlation preservation between pairs of module eigengenes across the two networks. Each column and row represented by consensus module eigengene with the saturation of red color showing adjacency according to the color legend.

Differential eigengene network analysis (Fig 7) was performed to address the comprehensive preservation of the correlation of consensus ME pairs of two stress-specific networks. Eigengene networks were constructed based on correlations between each pair of consensus MEs for evaluating preservation of modules and connectivity between cold and drought dataset. It was found that density, D (Preserv^Cold,Drought^) is 0.91, which indicate very high correlation preservation between all pairs of eigengenes across the two networks. The Consensus eigengenes (MEs) in the cold dataset were defined by two main groups, or meta-modules (Fig 7A). The first meta-module consisted of the C3 and C4 consensus modules (turquoise and yellow) and second meta-module contained C1 and C2 consensus modules (brown and blue). Remarkably, these 2 meta-modules were also approximated in the drought dataset (Fig 7B). Meta-modules in the cold and drought dataset indicated the following relationships: the first (turquoise and yellow) suggested a relationship among photosynthesis and defense response; the second (brown and blue) between response to abiotic stress and translation. This eigengene network analysis revealed that in addition to these four consensus modules being present in both the stress-specific dataset, the organization of these consensus modules was also highly preserved across the networks.

## Conclusion

The present study deals with meta-analysis of microarray studies related to drought and cold stress of *Arabidopsis thaliana*. This analysis was able to identify DEGs which include- DEGs already reported by individual studies and additionally, new DEGs which were overpass by individual studies. Thus, this approach magnifies the strength and sensitivity in the identification of vital stress response genes which may be overlooked by individual studies.

The comparative analysis of differential expression analysis and gene ontology enrichment of the two stresses revealed the existence of shared and unique components between cold and drought stress. It was found that several transcription factor families common in both the stresses regulates several common stress-responsive genes adhering to ABA-dependent pathway. The shared stress-responsive genes were found to be involved in ROS scavenging, stomatal movement etc. This helps the plant to reclaim the homeostatic state which was disturbed under the influence of both the stresses.

Gene co-expression network analysis also supported the findings of meta-expression analysis by revealing the existence of highly inter-correlated stress-specific and consensus modules with specific profiles of expression under drought and cold stress respectively. Altogether, the result from our study gives information about the common and unique biological and molecular behavior of the plant in response to various abiotic stresses which can be utilized for multiple stress response engineering.

## Supporting information

S1 Table Data sets used in meta-analysis and co-expression network generation. (XLSX)

S2 Table List of differentially expressed genes. (XLSX)

S3 Table Common differentially expressed genes in cold and drought stress. (XLSX)

S4 Table Comparison with transcriptome individual study literature. (XLSX)

S5 Table S5A Table. Comparison of drought and cold common differentially expressed genes with drought and cold common literature genes. S5B Table. Genes commonly up-regulated under both drought & cold stress in present analysis as well as mentioned in Kilian et al., 2006 study of cold & drought stress in *Arabidopsis*.
(XLSX)

S6 Table Comparison of frequencies of gene ontology terms for genes expressed under cold and drought stress. (XLSX)

S7 Table Number of transcription factors belonging to different transcription factor families in cold and drought stress DEGs list. (XLSX)

S8 Table Modules of each DEGs set along with their kTotal (whole network connectivity),kWithin (within module connectivity), MM (Module Membership) and p-values. (XLSX)

S9 Table List of co-expression modules found in each stress gene set. (XLSX)

S10 Table Comparison of drought modules against those detected by Shaik et.al. (2013). (XLSX)

S11 Table List of significant functional terms associated with genes in different co-expression modules. (XLSX)

S12 Table List of significant functional terms associated with genes in consensus modules. (XLSX)

S13 Table PRISMA checklist. (DOC)
